# Sudden death in a rat model of Parkinson’s disease

**DOI:** 10.6061/clinics/2021/e2974

**Published:** 2021-06-07

**Authors:** Fulvio Alexandre Scorza, Laís Damasceno Rodrigues, Leandro Freitas Oliveira, Antônio Carlos G. de Almeida, Josef Finsterer, Marcelo A. Moret, Giancarlo de Mattos Cardillo, Carla Alessandra Scorza

**Affiliations:** I Disciplina de Neurociencia, Escola Paulista de Medicina/Universidade Federal de Sao Paulo (EPM/UNIFESP), Sao Paulo, SP, BR; II Centro de Neurociencias e Saude da Mulher “Professor Geraldo Rodrigues de Lima”, Escola Paulista de Medicina, Universidade Federal de Sao Paulo (EPM/UNIFESP), Sao Paulo, SP, BR; III Laboratorio de Neurociencia Experimental e Computacional, Departamento de Engenharia de Biossistemas, Universidade Federal de Sao Joao del-Rei (UFSJ), Sao Joao del-Rei, MG, BR; IV Krankenanstalt Rudolfstiftung, Messerli Institute, Vienna, Austria; V Universidade do Estado da Bahia (UNEB), Salvador, BA, BR

In Parkinson’s disease (PD), extensive research using experimental models has been conducted to study different aspects of the disease to understand its etiology, pathology, molecular mechanisms, and potential therapeutic targets (1). Despite interesting debates in the literature, the available animal models are indispensable for the development of new concepts and ideas that can lead to novel protective or disease-modifying therapies (2). However, little contribution has been made in the understanding of sudden unexpected death in PD (SUDPAR).

PD, a very common age-related neurodegenerative disease, affects millions of people globally, has no cure, and is accompanied by high rates of premature death compared with the general population (3-5). SUDPAR has been identified as an important cause of death in PD (3-5). Didactically, SUDPAR is defined as the unexpected death of a patient with PD without any satisfactory cause of death, as determined by an autopsy (3-5). Although there are no epidemiological studies that precisely demonstrate the possible distribution of SUDPAR in the main research centers for movement disorders, a general analysis of relevant studies on SUDPAR has shown that an average of 14% of patients with PD die suddenly (3). Additionally, several risk factors may be directly associated with SUDPAR such as age at onset, disease duration, sex, the severity of motor involvement, sleep disorder, concomitant cardiac and pulmonary disease, and polypharmacy (3,5). Although the mechanisms of death in SUDPAR are under discussion, there are strong indications that cardiac abnormalities and autonomic dysfunction play a possible “direct” role in SUDPAR since structural and functional abnormalities of the heart are found in approximately 60% of the patients with PD (3,5). Considering these data, our research group is convinced that adequate animal models are currently indispensable for studying the cause and progression of PD, as well as the possible mechanisms and risk factors related to SUDPAR.

In this context, the 6-hydroxydopamine (6-OHDA) model continues to constitute a classic animal model of PD in rats that has contributed to our current knowledge about motor and biochemical dysfunctions in PD (6). Importantly, several studies have also provided significant evidence that the 6-OHDA animal model can also be used to assess cardiovascular and autonomic changes, mimicking those found in patients with PD (7-10). Considering the relevance of this model, we detected a case of a Wistar rat subjected to 6-OHDA that died suddenly, and the event was recorded during routine electrocardiogram (ECG) monitoring. For this, we used an adult male Wistar rat that underwent stereotactic surgery to establish the 6-OHDA model and place the cardiac electrodes. After the *in vivo* procedures, the animal brain was removed for immunohistochemical analysis to verify the effectiveness of the model (Figure 1). In this context, we recorded the following. 

In brief, on day 7 after intracerebral administration of 6-OHDA concomitant with implantation of cardiac electrodes, the animal presented normal sinus rhythm with a heart rate (HR) of approximately 360 bpm (Figures 2A and 2B). On experimental day 10, the rat showed an increase in HR of approximately 480 bpm (Figure 2C) with ventricular extrasystoles (Figure 2D). Three hours after the beginning of the first extrasystole, an episode of ventricular tachycardia of approximately 540 bpm was recorded (Figure 2E) that evolved into ventricular tachycardia with sinus blocks (Figure 2F). Five hours after the first cardiovascular changes, the animal presented *Torsades de Pointes* (Figure 2F) progressing to isoelectric electrographic activity (Figures 2G and 2H). According to the immunohistological data (Figures 2I and 2H) on day 10 after the 6-OHDA administration, a statistically significant reduction was detected in the dopaminergic neurons in the *substantia nigra* on the side ipsilateral to the administration of the neurotoxin when compared to its contralateral side (*p*<0.001) (Figure 2K).

Given these results, some considerations are relevant to this scenario. To the best of our knowledge, this is the first report to provide evidence of sudden death in an animal model of PD during ECG monitoring. Moreover, rats are the most commonly used species in experimental cardiac physiology and electrophysiological studies (11). Furthermore, it is well established in the literature that ECG is a valuable tool for the diagnosis and monitoring of cardiac arrhythmias and conduction disturbances (11). Accordingly, a series of clinical trials have investigated the relationship between clinical features and ECG parameters in patients with PD (3,12-14). These studies demonstrated that several ECG parameters reflect autonomic dysfunction or disease progression, strongly suggesting that clinicians should pay more attention to ECG abnormalities when treating patients with PD (3,12-14). From an experimental point of view, several authors have also described cardiovascular changes in PD animals, which could be directly linked to cases of sudden death (7-10). Thus, a recent study conducted by our research group showed that animals subjected to 6-OHDA showed a reduction in HR and greater variability of heartbeats using the *Poincaré plot* technique (10), which allows us to gain new insights into the dynamics of cardiac neural mechanisms (15). In light of these findings, the results of the present study demonstrate that the use of ECG monitoring in animal models of PD may demonstrate important cardiac abnormalities and may register sudden fatal events in PD.

As described previously, cardiac abnormalities and autonomic dysfunction seem to be the most important issue in SUDPAR (3). Thus, it is evident that identifying individuals at risk of SUDPAR is the best way to prevent it. Considering this approach, it has been proposed that strategies for routine cardiovascular screening (ECG, Holter monitoring) should be applied to further reduce the frequency of SUDPAR (3,5). In these terms, the results of this study corroborate previous clinical investigations suggesting that routine ECG monitoring is a valuable, inexpensive, simple, and reproducible marker of sudden cardiac death (15).

Another important factor to be discussed is age; aging remains the biggest risk factor for idiopathic PD (16). It has been demonstrated that aging triggers a cascade of stressors within the *substantia nigra*, which essentially weakens dopaminergic neurons and their ability to respond to further insults that are seen as part of the disease process (16). From an experimental perspective, this issue remains a matter of debate. The use of rodents of variable age has been shown to impact data quality in many areas of scientific research (17). However, in PD research, most studies did not use aged animals (18). Specifically, regarding the 6-OHDA-induced rat model of PD, it has been established that the best strategy would be to perform lesional surgery when animals are young and develop the disease phenotype with aging, which might result in a decreased animal death rate (18). Considering the age-related criteria for designing experiments with the 6-OHDA model, the case of sudden death described in this study is in accordance with the proposals established in the literature.

Another important point is that independent of the site of injection, the 6-OHDA-induced dopamine depletion appears to be a valuable model to investigate clinical PD manifestations and gain more insight into the systemic abnormalities observed in this neurodegenerative disease (7-10,19,20). Both the unilateral and bilateral 6-OHDA models have their advantages and disadvantages (20,21). However, none of the currently available animal models can completely reproduce the clinical characteristics of PD (21), although the unilateral 6-OHDA model used in the present study remains the most widely used model for experimental studies of PD in rats (22,23). Importantly, the immunohistochemical and cardiovascular data demonstrated here are consistent with previous studies of Parkinsonism in the unilateral 6-OHDA animal model of PD, suggesting that the rats were adequately lesioned (7,8,10,19,22).

Overall, there were several limitations of this study. Although the techniques applied are the most practiced, the model induced by 6-OHDA does not include all PD symptoms and does reproduces the main histological abnormalities involved in PD (24). Furthermore, changes in HR and behavioral indexes in the 6-OHDA model should be quantitatively monitored in rats using HR telemetry and video systems. In addition, the number of animals (n=01) was insufficient to make accurate statements in SUDPAR. However, SUDPAR is a rare phenomenon that is difficult to diagnose and is rarely reported (7). Thus, the case of sudden death in our rat model might reflect the features of sudden death in humans. As a final step of the experiments, a necropsy should have been performed immediately after the animal’s sudden death. In general, more translational studies are required to study the prevalence of SUDPAR and explore possible predisposing factors and causal mechanisms for this condition.

In conclusion, the present study reported a case of SUDPAR in a rat, 10 days after administration of 6-OHDA to the striatum, suggesting the usefulness of the 6-OHDA animal model to better understand the cardiovascular procedures associated with PD and SUDPAR.

## Figures and Tables

**Figure 1:**
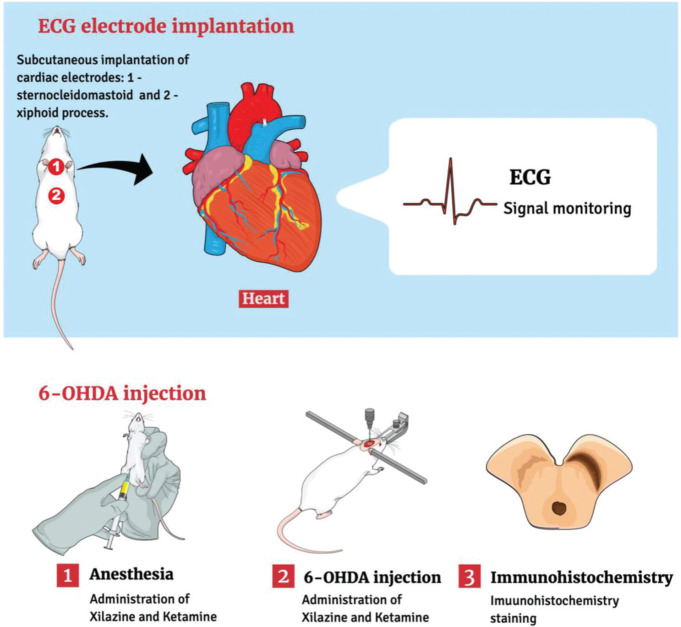
Schematic representation of the experimental procedures 6-OHDA, 6-hydroxydopamine; ECG, electrocardiogram.

**Figure 2:**
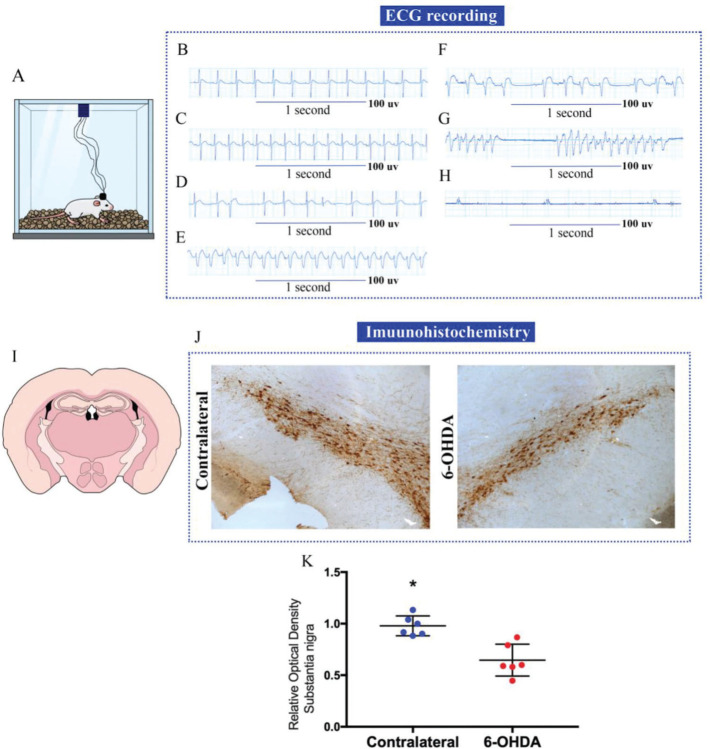
Electrocardiogram (ECG) recordings from a Wistar rat submitted to 6-hydroxydopamine (6-OHDA) that died of sudden unexpected death in Parkinson’s disease (SUDPAR). **(A)** Animal in the Faraday cage for ECG recording; **(B)** Normal ECG, 3 days before death, with heart rate (HR) of 360 bpm; **(C)** ECG recording on the day of death with 480 bpm HR; **(D)** Evolution of ECG with the presence of ventricular extrasystoles, **(E)** ECG with 540 bpm ventricular tachycardia; **(F)** ECG with ventricular tachycardia and sinus block; **(G)** *Torsades de Pointes*, **(H)** ECG with absent electrical activity; **(I)** Image showing a slice of the striatum region of the animal; **(J)** Slice of the *substantia nigra* region of the brain showing dopaminergic neurons marked by the tyrosine hydroxylase antibody; **(K)** Analysis of the optical density of immunohistochemistry in the *substantia nigra* region. The asterisk (*) indicates a statistically significant difference between the two sides of the *substantia nigra* (*p<0.05). The figure is created in the Mind the Graph platform http://www.mindthegraph.com under creative commons license CC community as “attribution share-alike 4.0 licensing”: https://creativecommons.org/licenses/by/4.0/.
